# Editorial: The role, pathophysiology, and clinical benefit of collateral circulation in acute and chronic ischemic stroke

**DOI:** 10.3389/fneur.2023.1281009

**Published:** 2023-10-06

**Authors:** Andrea M. Alexandre, Alessandro Pedicelli, Aldobrando Broccolini

**Affiliations:** ^1^UOSD Neuroradiologia Interventistica, Fondazione Policlinico Universitario A. Gemelli IRCCS, Rome, Italy; ^2^UOC Neurologia, Fondazione Policlinico Universitario A. Gemelli IRCCS, Rome, Italy; ^3^Università Cattolica del Sacro Cuore, Rome, Italy

**Keywords:** stroke, ischemic stroke, collateral circulation, endovascular thrombectomy, rt-PA

Ischemic stroke remains one of the leading causes of mortality and disability worldwide, posing an urgent challenge for medical science. The intricate interplay between cerebral perfusion and neural function underscores the need for comprehensive insights into stroke pathophysiology. Recent advancements in neuroimaging and therapeutic interventions have rekindled interest in collateral circulation, a natural compensatory mechanism that assumes paramount importance in the context of ischemic stroke (1, 2). This Research Topic delves into the multifaceted significance of collateral circulation in cerebral ischemia, shedding light on its intricate mechanisms, clinical implications, and potential avenues for therapeutic interventions (3, 4).

Collateral circulation transcends mere anatomical pathways; it embodies a dynamic physiological response, and it refers to the intricate network of secondary vessels that can reroute blood flow to ischemic brain regions when the primary arterial supply is compromised. Notably, the extent and efficiency of collateral circulation vary greatly among individuals, partially contributing to the heterogeneous outcomes observed in ischemic stroke cases (5, 6).

The understanding of cerebral collateral circulation, starting from anatomy, its development, and its role in acute or chronic ischemic stroke is essential when dealing with this pathology. These aspects are useful both in common clinical practice as well as in clinical research to increase the chance of a better outcome after a cerebral ischemic event (7–10).

In this Research Topic, Xiufu et al. present an interesting “*Analysis of the influencing factors of early neurological improvement after intravenous rt-PA thrombolysis in acute anterior circulation ischemic stroke”*. They demonstrate the effect of rt-PA intravenous thrombolysis within the time window in patients with acute anterior circulation ischemic stroke. Moreover, they argue that diabetes is the most important factor affecting the clinical outcomes of acute anterior circulation ischemic stroke in patients, showing early neurological improvement after intravenous thrombolysis with rt-PA.

In their research on how “*Cerebral blood flow velocity progressively decreases with increasing levels of verticalization in healthy adults. A cross-sectional study with an observational design*” Deseoe et al. show that in healthy adults there is a significant, progressive drop in CBFV with progressive levels of verticalization, using a dynamic protocol with 3–5 min maintaining single positions. Mean differences in CBFV between consecutive positions of head-up tilt ranged from −0.6 cm/s or −1.4% to −2.2 cm/s or −5.2%. At the same time, there was no relevant change in CBFV from baseline to −5°. This study was conceived as the starting point for further investigations in patients with impaired regulation of CBF. It is known that patients show impairments in the regulation of CBF after stroke and that these impairments can be predictive of worse functional outcomes.

A challenging endovascular treatment: “*Case report: Flow changes in routes of collateral circulation in patients with LVO and low NIHSS: a point favor to treat*” by dos Santos Neto et al. highlights the importance of collateral flow in predicting stroke outcomes and response to treatment. Patients with poor collaterals may be more susceptible to early neurological deterioration and may benefit from early intervention, even with a low NIHSS score. In this report, the patient had compensatory collateral flow from the circle of Willis but presented a neurological worsening and failure in collateral flow, suggesting a need for urgent treatment. This report focuses on the importance of close monitoring of collateral flow and response to treatment in patients with LVO stroke. An intensive transcranial Doppler monitoring strategy could be useful in identifying patients who may benefit from endovascular thrombectomy. Transcranial Doppler can provide real-time information on changes in blood flow velocity and collateral flow patterns, allowing for early detection of neurological deterioration and prompt intervention.

Considering the patient holistically in the case of the ischemic cerebral stroke means also evaluating the psychological aspect associated with the disease, as demonstrated in the work by Motolese et al. on “*The role of neurophysiological tools in the evaluation of ischemic stroke evolution: a narrative review*”, in which they evaluate how neurophysiological tools might still play a role in the evaluation of stroke, even in the “Imaging is brain” era. Neurophysiological tools are of great value for capturing these changes over time thanks to the excellent time resolution, allowing the longitudinal evaluation till the chronic phase, even if the methodological heterogeneity of the literature has limited the diffusion of techniques such as EEG or TMS in daily practice.

Neurophysiology and neuroimaging should be then considered as complementary tools exploring the same event from two different perspectives. This also regards the viability of penumbra brain tissue during stroke and, indirectly, the blood flow status. More studies are warranted to get a better insight into stroke pathophysiology. This is of critical importance for developing a tailored rehabilitation approach.

On the other hand, the study “*Determinants of cerebral collateral circulation in acute ischemic stroke due to large vessel occlusion*” by Sperti et al. evaluates a large cohort of patients (520) with acute ischemic stroke and large vessel occlusion of anterior circulation potentially eligible for endovascular treatment, in which the authors investigated the associations between clinical factors and collaterals and tested independent associations with logistic (good vs. poor collaterals) and ordinal (collateral grade grouped, Menon 0–2, 3, 4–5) regression analysis adjusting for age, sex, stroke severity, and onset to CT time. Their findings suggest that risk factors and demographics do not influence the development of collateral circles, except for a negative relation with previous ischemic events, assuming NIHSS as its surrogate.

Finally, Xu et al. in their “*Quantitative assessment of collateral time on perfusion computed tomography in acute ischemic stroke patients*” divide the parasagittal region of the ischemic hemisphere into six pial arterial zones according to pial branches of the middle cerebral artery. Within the 85 arterial zones with collateral vessels, the receiver operating characteristic analysis was performed to derive the optimal collateral time threshold for fast collateral flow on perfusion computed tomography. The optimal collateral time threshold for fast collateral flow on perfusion computed tomography was a delay time of 4.04 s.

## Author contributions

AA: Conceptualization, Investigation, Writing—original draft, Writing—review and editing. AP: Writing—original draft, Writing—review and editing. AB: Writing—original draft, Writing—review and editing.
